# Regulation of the Expression of Chaperone gp96 in Macrophages and Dendritic Cells

**DOI:** 10.1371/journal.pone.0076350

**Published:** 2013-10-16

**Authors:** Lutz Wolfram, Anne Fischbeck, Isabelle Frey-Wagner, Kacper A. Wojtal, Silvia Lang, Michael Fried, Stephan R. Vavricka, Martin Hausmann, Gerhard Rogler

**Affiliations:** Division of Gastroenterology and Hepatology, University Hospital of Zurich, Zurich, Switzerland; INSERM, France

## Abstract

The chaperone function of the ER-residing heat shock protein gp96 plays an important role in protein physiology and has additionally important immunological functions due to its peptide-binding capacity. Low amounts of gp96 stimulate immunity; high quantities induce tolerance by mechanisms not fully understood. A lack of gp96 protein in intestinal macrophages (IMACs) from Crohn`s disease (CD) patients correlates with loss of tolerance against the host gut flora, leading to chronic inflammation. Since gp96 shows dose-dependent direction of immunological reactions, we studied primary IMACs and developed cell models to understand the regulation of gp96 expression. Induction of gp96-expression was higher in *in vitro* differentiated dendritic cells (*i.*v.DCs) than in *in vitro* differentiated macrophages (*i.v.*MACs), whereas monocytes (MOs) expressed only low gp96 levels. The highest levels of expression were found in IMACs. Lipopolysaccharide (LPS), muramyl dipeptide (MDP), tumour necrosis factor (TNF), and Interleukin (IL)-4 induced gp96-expression, while IL12, IL-17, IL-23 and interferon (IFN)-γ were not effective indicating that Th1 and Th17 cells are probably not involved in the induction of gp96. Furthermore, gp96 was able to induce its own expression. The ER-stress inducer tunicamycin increased gp96-expression in a concentration- and time-dependent manner. Both ulcerative colitis (UC) and CD patients showed significantly elevated gp96 mRNA levels in intestinal biopsies which correlated positively with the degree of inflammation of the tissue. Since gp96 is highly expressed on the one hand upon stress induction as during inflammation and on the other hand possibly mediating tolerance, these results will help to understand the whether gp96 plays a role in the pathophysiology of inflammatory bowel disease (IBD).

## Introduction

The intestinal mucosa constitutes the primary anatomic and immunological barrier to the gut lumen preventing uncontrolled entry of food and commensal antigens into the body. The innate immune system of the intestine has evolved to benefit both the host and the microflora by keeping homeostasis of the epithelial cell tissue layer and tolerance versus the gut bacteria, while being able to elicit specific responses towards invading pathogens. Any disturbance in this host-commensal relationship may imbalance the innate immune signalling leading to intestinal inflammation in the genetically susceptible host. Inflammatory bowel disease (IBD) is regarded to be a consequence of such a disturbance of intestinal barrier and innate immune functions [1], [2].

A central component of the intestinal innate immune system are intestinal macrophages (IMACs) representing the largest population of macrophages in the body [3]–[5]. They originate from immigrating monocytes. Upon their differentiation in the mucosal environment IMACs downregulate the expression of many pattern recognition receptors (PRRs) and co-stimulators leading to a tolerogenic phenotype in healthy individuals [6], [7]. In contrast to many cell surface receptors [6], [8] and proteasomal components important for antigen presentation (unpublished data), which also undergo downregulation in IMACs, the glycoprotein (gp) 96 is strongly induced during the differentiation of monocytes to IMACs [9].

Gp96 is a multifunctional endoplasmic reticulum (ER)-resident protein that has recently gained interest in being a mediator between innate and adaptive immune reactions [10]–[11]. Furthermore, recent data connect a loss of gp96 to a perturbance in ER stress signalling [12]. ER stress occurs upon accumulation of mis- or unfolded proteins in the ER [13]–[15]. ER stress has recently been speculated to be a potential initiator of intestinal inflammation [16], while it formerly has only been observed concomitantly with intestinal inflammation [17]–[19].

Originally gp96 was identified as a heat shock protein (HSP) [20]. Like other HSPs it is phylogenetically highly conserved in all prokaryotes and eukaryotes [21]–[25]. What is relevant for immunological reactions is the peptide binding capacity of gp96 in the ER where the peptides originate from intracellular degradation of proteins [11], [26]. Gp96-peptide-complexes released upon stress and necrosis can lead to different immunological outcomes. Specific uptake [27]–[30] by antigen presenting cells triggers cross-presentation [30] of the peptide on major histocompatibility complex (MHC) I conferring either immunity or tolerance towards the cells where gp96 had been isolated from [10], [31]–[33]. Speculation about direct interaction of gp96 with toll-like receptors has been linked to the activation of macrophages (MΦs) and dendritic cells (DCs) [34], [35], although this has been under debate due to the possibility of contaminating lipopolysaccharides (LPS) in the applied recombinant gp96 [36], [37]. Gp96 may act as an efficient adjuvant to initiate immune responses.

In this context it is striking that gp96 was not immunohistochemically detectable in IMACs from Crohn's Disease (CD) patients, whereas it is abundantly expressed in tolerogenic IMACs of healthy individuals [9]. Since the presence of gp96 protein coincides with healthy and inflamed regions in the colon, we investigated the regulation of expression in IMACs and in *i.*v.DCs and *i.v.*MACs, both basal and after stimulation with pro-inflammatory cytokines, bacterial cell envelope components as well as purified human gp96.

## Results

### The expression of gp96 is induced upon differentiation into IMACs, *i.*v.MACs and *i.*v.DCs

**Figure 1:**
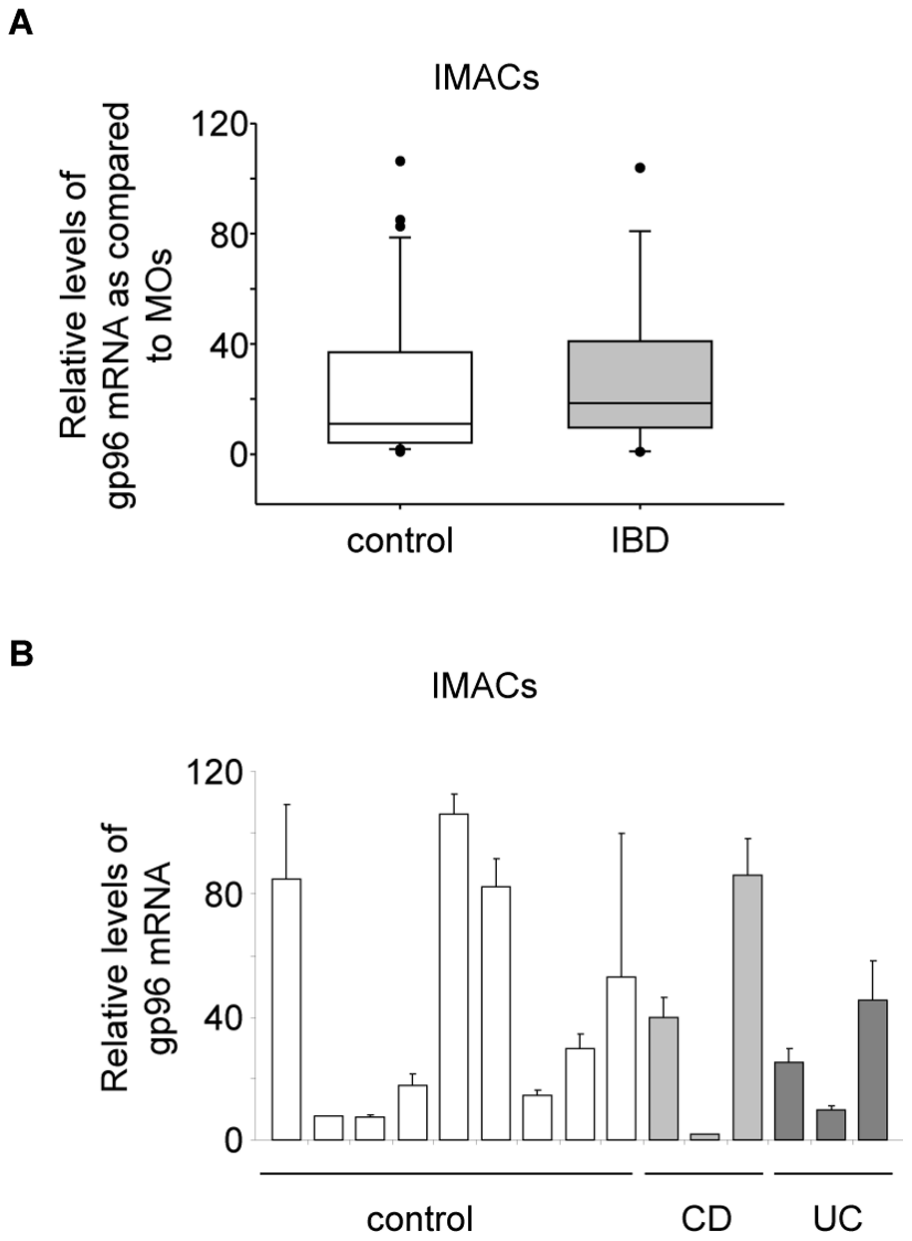
mRNA expression of gp96 in IMACs from IBD and control patients. Control patients comprised 31 with cancer, 3 with fistulae, 1 with volvulus, and 1 healthy patient, 8 with diverticulitis, and 3 with diverticulosis. IBD patients comprised 6 CD and 5 UC patients. (A) Average expression (vs. GAPDH) from IMACs of control (n = 47) and IBD patients (n = 11). Error bars represent the standard deviation of the mean of all analysed patients. (B) Selected expression levels of IMACs from single patients. The expression in MOs was used as a reference and set to 1. Error bars represent the standard deviation of a single experiment measured in triplicate.

**Figure 2:**
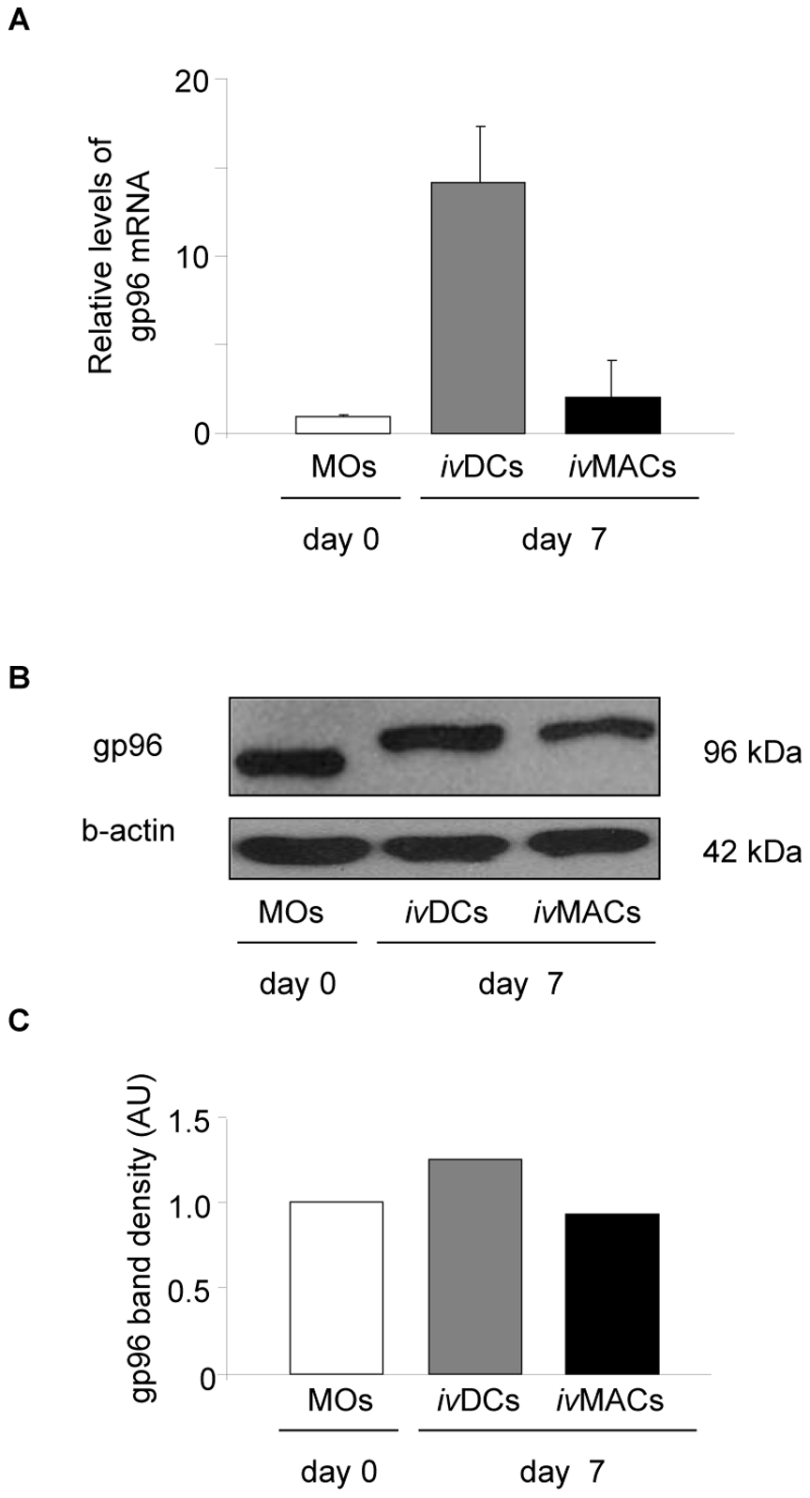
Regulation of gp96 expression during differentiation of MOs to *i.*v.DCs and *i.*v.MACs. (A) Relative mRNA levels of gp96 (vs. GAPDH) by RT-PCR. Results are representative for three experiments in total. A typical result is shown where the basal level in MOs is set as a reference. Error bars represent the standard deviation of a single experiment measured in triplicate. (B) Western blot analysis of gp96. (C) Densitometric analysis. AU: arbitrary units, kDa: Kilodalton.

In the gut mucosal environment gp96 mRNA expression was induced to high levels (between 30 and 100-fold) in IMACs (Fig. 1) in comparison with their progenitor cells monocytes showing the lowest expression of the tested cell types. There was overall no significant difference between expression levels of gp96 in IMACs from control and IBD patients (Fig. 1A, B). There was no correlation between expression levels of gp96 and patient age or gender. After one week of *in vitro*-differentiation an induction of mRNA expression was detected (up to 15-fold) for *i.*v.DCs but only a moderate induction for *i.*v.MACs was observed (Fig. 2A). Analysis of protein levels of both cell types confirmed this observation (Fig. 2B, C), albeit to a much lesser extent than on mRNA level (only about 1.5-fold increase in *i.*v.DCs, Fig. 2C).

### TNF stimulation leads to an upregulation of gp96 expression

In order to investigate the regulation of gp96 expression, we challenged the cells with stimuli present in the mucosal environment and known to initiate immune and/or inflammatory responses. These were the cytokines TNF, IFN-γ, granulocyte-macrophage colony stimulating factor (GM-CSF), IL-4, IL-12, IL-17, and IL-23. During a first course of experiments we found consistently that the highest levels of gp96 mRNA were induced after two hours of stimulation, independently of the stimulus. Therefore, in the following experiments samples for RNA isolation were always taken after two hours of stimulation.

**Figure 3:**
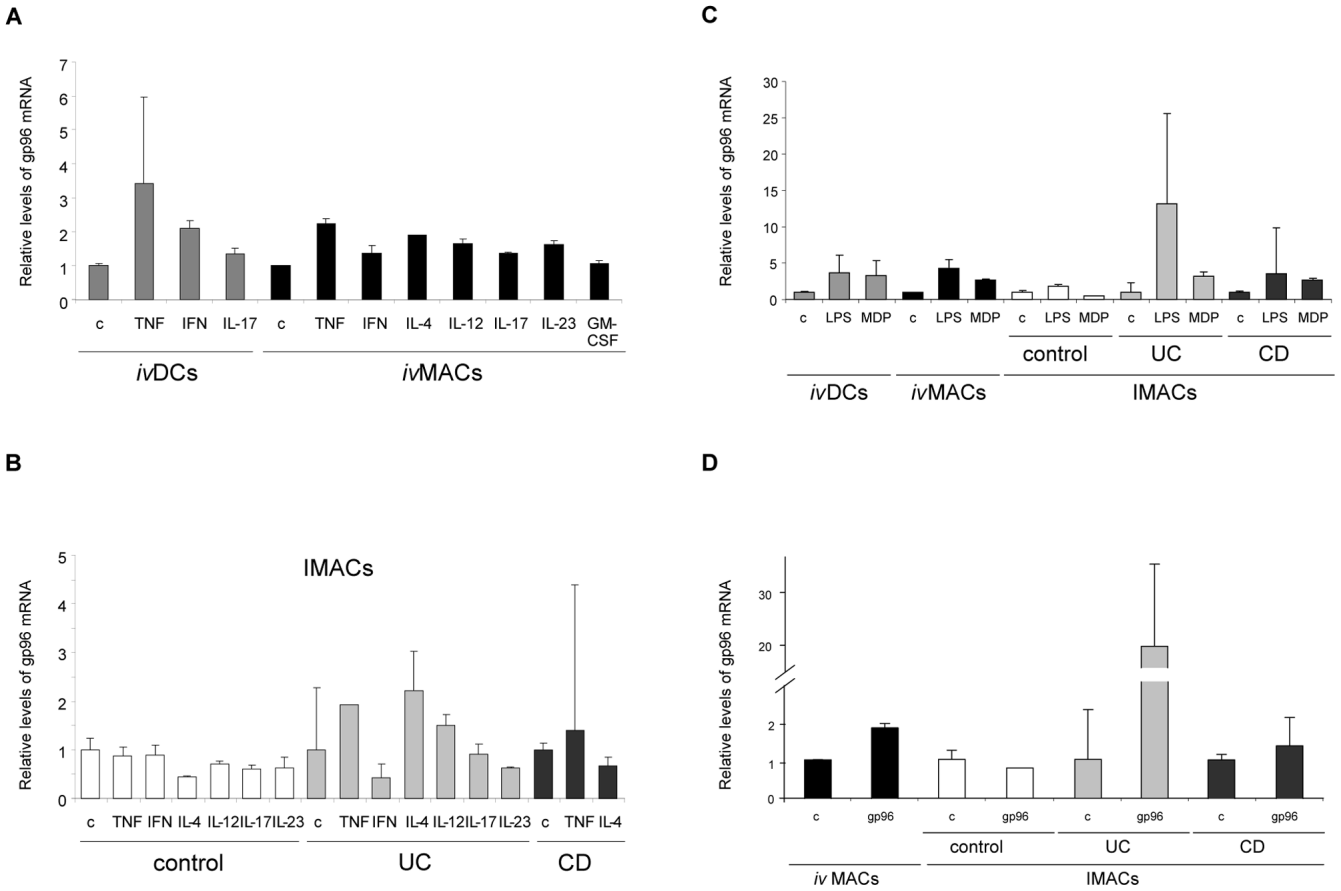
Stimulus-dependent expression of gp96 mRNA in human innate immune cells. Relative mRNA levels of gp96 are shown (vs. GAPDH/β-actin) in *i.*v.DCs, *i.*v.MACs and IMACs from control, UC, and CD patients. Cells were stimulated for 2 h. Results are representative for three experiments. Error bars represent the standard deviation of a single experiment measured in triplicate. (A) Stimulation of *i.*v.DCs and *i.*v.MACs with cytokines. (B) Stimulation of IMACs with cytokines. (C) Stimulation of *i.*v.DCs, *i.*v.MACs and IMACs with bacterial envelope components. (D) Stimulation of *i.*v.MACs and IMACs with gp96 protein. c: unstimulated control; TNF: tumour necrosis factor, IFN-γ: interferon-gamma, IL: interleukin IL-12, IL-17, IL-23 (100 ng/mL each); GM-CSF: granulocyte-macrophage-colony stimulating factor (50 ng/mL); IL-4 (5 ng/mL); LPS: lipopolysaccharide, MDP: muramyl-dipeptide (10 ng/mL each); gp96: glycoprotein 96 (10 µg/mL).

Stimulation of *i.*v.DCs and *i.*v.MACs (Fig. 3A) as well as IMACs from control and IBD patients (Fig. 3B) with cytokines revealed that only some were able to induce gp96 expression. Among all tested cytokines TNF was the most potent: apart from irresponsive IMACs of control patients all cell types increased the synthesis of gp96 mRNA between 3.5- (*i.*v.DCs) and 2-fold (*i.*v.MACs and IMACs from IBD patients), respectively, while IFN-γ was able to induce higher levels only in *i.*v.DCs (2-fold). Upon incubation with IL-4 *i.*v.MACs and IMACs from UC patients synthesized 2- and 2.5-fold more gp96 mRNA, respectively. IL-12 induced a 2-fold rise only in IMACs from UC patients. The other cytokines IL-17, IL-23 and GM-CSF did not regulate expression of gp96 under the conditions studied.

### The bacterial cell envelope components LPS and MDP induce increased gp96 mRNA levels

IMACs from IBD patients, *i.*v.DCs and *i.*v.MACs showed a high induction of gp96 mRNA after stimulation with the bacterial cell envelope components LPS and MDP. In contrast the hardly responsive IMACs from non-IBD control patients were only slightly increasing expression after stimulation with LPS (Fig. 3C). *i.*v.DCs and *i.*v.MACs were observed to be similarly responsive: LPS induced mRNA synthesis about 4-fold, MDP approximately 3-fold. Both components induced about equal levels of gp96 mRNA in IMACs from CD patients, while IMACs from UC strongly increased gp96 mRNA synthesis after stimulation with LPS (13-fold). IMACs from non-IBD patients only slightly changed gp96 mRNA levels upon stimulation.

### Gp96 induces its own expression

The results of stimulation experiments with purified human gp96 are shown in Fig. 3D. Self-induction of gp96 was observed in *i.*v.MACs (about 2-fold) and IMACs from IBD patients. High expression levels were detected in IMACs from UC patients (19-fold). In IMACs from CD patients gp96 mRNA expression was only slightly induced (about 1.5-fold). Again IMACs from non-IBD patients were not responsive.

### ER-stress induces gp96 expression in cell lines and primary cells

**Figure 4:**
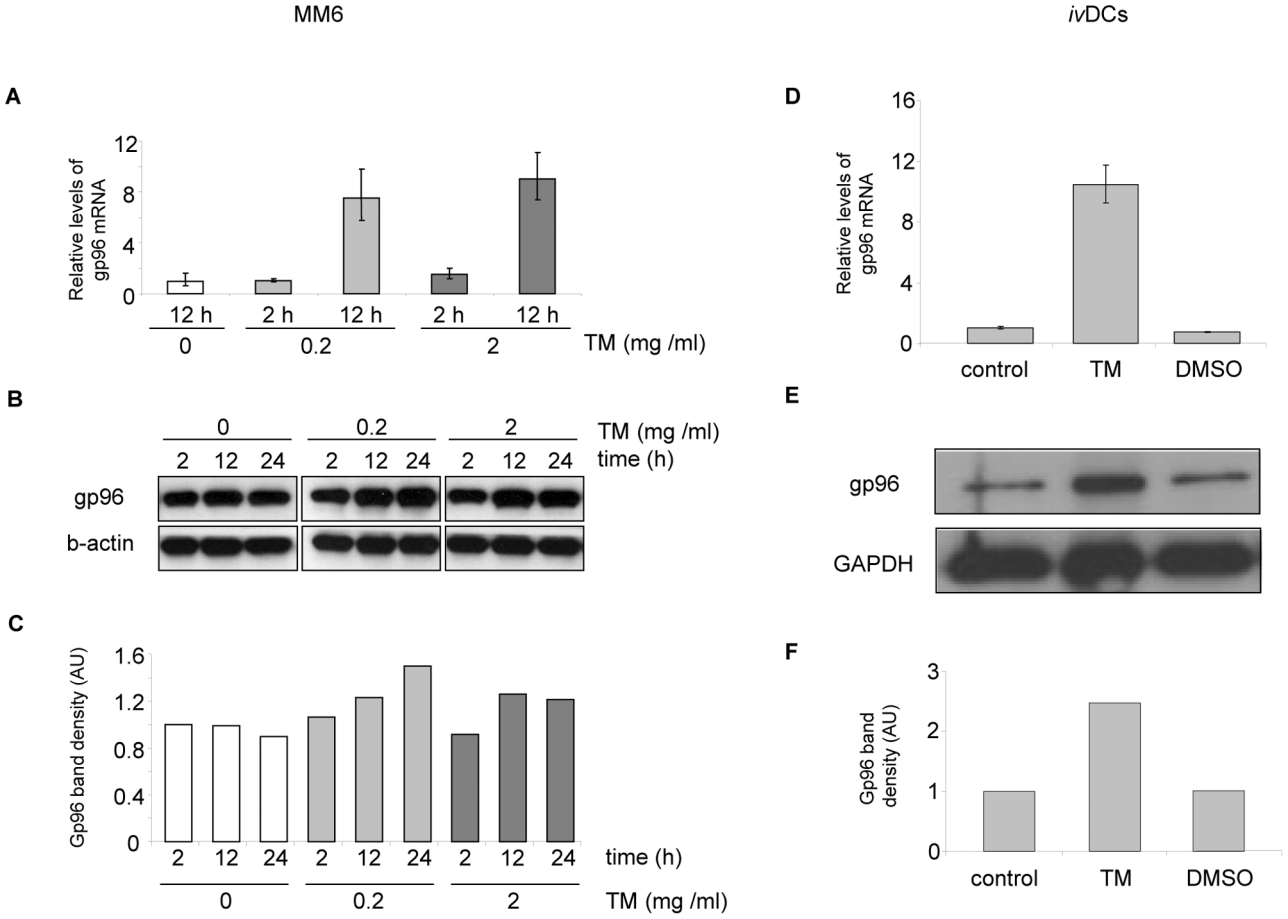
ER stress induction of gp96 in MM6 and *i.*v.DCs. Panels A–C show the results for MM6 after 2, 12, and 24 hours of incubation with tunicamycin, panels D-F for *i.*v.DCs after 24 hours. Results are representative for three experiments. Three concentrations of tunicamycin were used to induce ER-stress in MM6 cells: 0, 0.2, and 2 µg/mL. (A, D) Relative mRNA levels of gp96 (vs. β-actin). Error bars represent the standard deviation of a single experiment measured in triplicate. (B, E) Western blot analysis of gp96. (C, F) Densitometric analysis. AU: arbitrary units.

The well-known inducer of ER stress tunicamycin was tested to increase the expression of gp96 in MM6 cells and *i.*v.DCs. After 12 hours of stimulation with 0.2 µg/mL tunicamycin, gp96 mRNA levels were induced 7.5-fold in MM6 cells (Fig. 4A), 2 µg/mL of tunicamycin increased the level 9-fold. After 2 hours only a slight induction of gp96 mRNA levels was observed. In *i.*v.DCs the induction of gp96 mRNA was 10-fold after 24 h with 2 µg/mL of tunicamycin (Fig. 4D). In comparison gp96 protein levels upon incubation with tunicamycin increased 1.2- to 2.5-fold, depending on the incubation time, the concentration and the cell type (Fig. 4B, E, F). Interestingly the lower concentration of tunicamycin was gradually inducing gp96 synthesis over 24 h, while the higher concentration reached maximal induction already after 12 h (Fig. 4C).

### Expression of gp96 is elevated in biopsies from colon and terminal ileum of IBD patients

**Figure 5:**
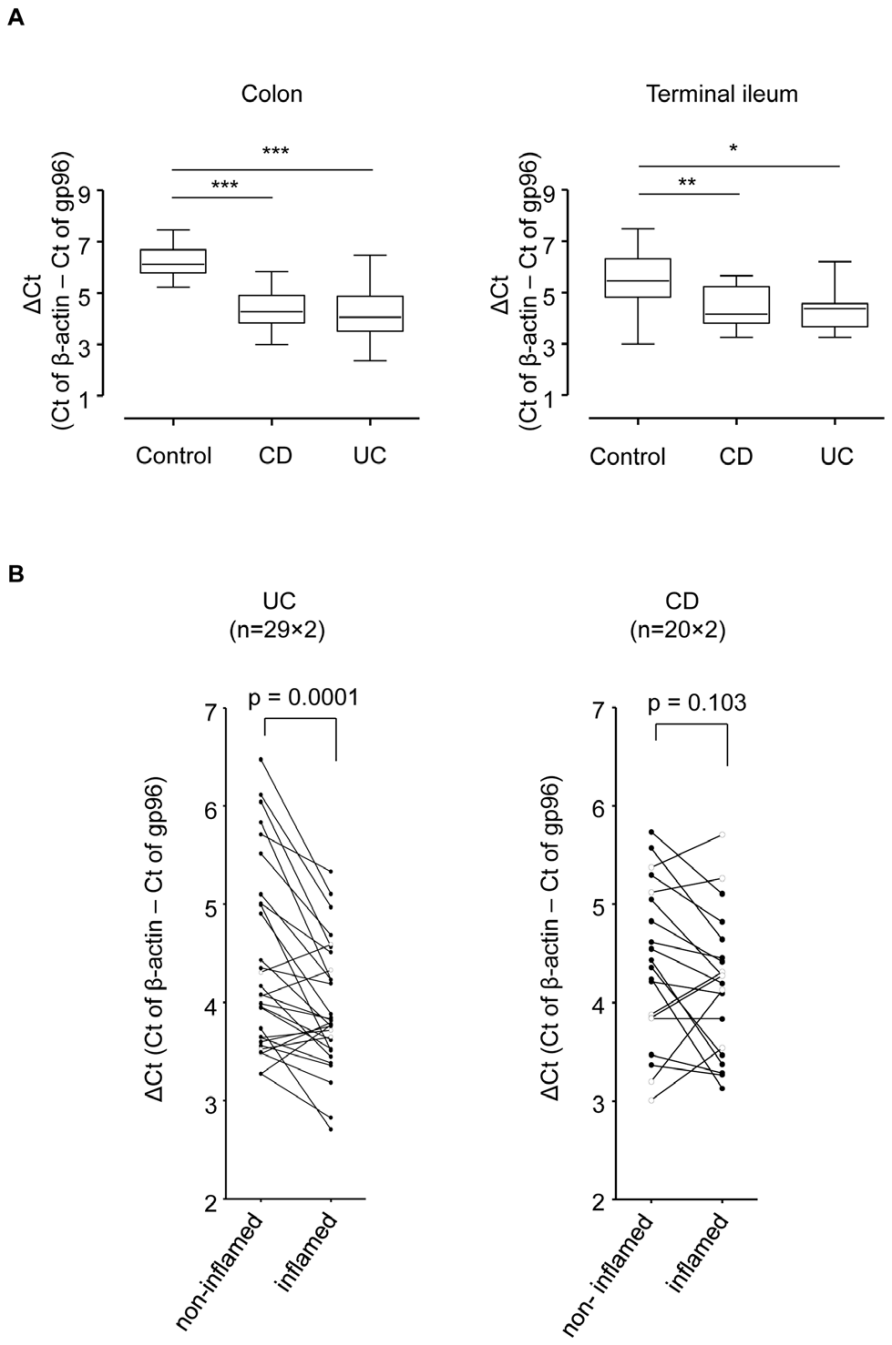
Analysis of gp96 mRNA in intestinal biopsies from IBD and control patients. (A) Relative mRNA levels of gp96 (vs. β-actin) in biopsies from colon and terminal ileum of control and IBD patients. (B) Paired analysis of gp96 expression levels within individual patients (involved vs. non-involved). Error bars represent standard error of the mean. * p<0.05, ** p<0.001, *** p<0.0001.

In intestinal biopsies from colon and terminal ileum gp96 mRNA levels were significantly elevated in IBD patients suffering from UC or CD as compared to control patients (Fig. 5A). A paired analysis of non-inflamed versus inflamed regions within individual patients confirmed the correlation between inflammatory status of the intestinal tissue biopsy and elevated gp96 mRNA levels in UC, and to some extent in CD, but in case of the latter the observed changes were not significant (Fig. 5B). We could not find any direct impact of IBD-related medication. However, it might be due to the limited number of patients that we did not find any pattern that was associated with the applied medication.

## Discussion

In this study we presented data on mRNA levels of the immune-relevant HSP gp96 in innate immune cells. The regulation of expression of immuno-relevant HSPs has not been fully understood yet. It is well known though that they are up-regulated in stress situations like e. g. heat shock, ER stress and hypoxia [24], and also during the differentiation of immune cells like e. g. IMACs [9]. Accordingly we observed a high induction of gp96 mRNA expression in our model of *i.*v.DCs. A lower induction was detected in *i.*v.MACs. IL-4 used in the differentiation process for *i.*v.DCs was able to induce an increased expression of gp96 also in *i.*v.MACs. [38] Additionally we clearly showed up-regulation of gp96 by the ER-stress inducer tunicamycin in the monocytic cell line MM6. This reflects the function of gp96 as a chaperone on the one hand (up-regulation during ER-stress) and as a mediator of immune responses on the other hand (up-regulation during differentiation of immune cells). The increase in expression of chaperones during ER-stress has been well characterized by three different unfolded protein response (UPR) pathways involving inositol-requiring enzyme (IRE) 1, protein kinase RNA-like ER kinase (PERK), and activating transcription factor (ATF) 6 [15]. However, what triggers the high induction of gp96 protein during the differentiation of IMACs in healthy individuals still needs to be elucidated. In comparison to IMACs *i.*v.MACs in our differentiation model expressed only slightly higher gp96 levels, although both develop from monocytes. Candidates involved in the regulation of gp96 expression in immune cells might be Ets repressor complexes that were described to arrest the cell cycle during terminal differentiation of monocytes into macrophages [39]. The observed lack of gp96 protein in CD-IMACs is most probably post-transcriptionally regulated, since mRNA levels were almost unchanged compared to anergic control IMACs [9].

Identifying regulators of expression of gp96 mRNA levels is a first step to clarify the mechanism(s) involved in the change of gene activity. Increased levels of gp96 were induced in cells challenged with pro-inflammatory stimuli as TNF, the bacterial cell envelope components LPS and MDP, additionally with gp96 protein itself and weakly with IL-4. This hints to TLRs, TNF-, and gp96-receptors as putative starting points for the activation of gp96 gene expression in innate immune cells. No induction was observed after stimulation with IFN-γ, IL-12, IL-17, and IL-23 usually found in inflamed mucosa of IBD patients in elevated concentrations [1], [2], [40]. The set of non-inducing cytokines seemed to exclude Th1 (typically secreting IFN-γ [41], [42]) and Th17 (secreting IL-17 [40]) cells from the transcriptional up-regulation of gp96 in innate immune cells.

The cytokine TNF and LPS and MDP – altogether pro-inflammatory – were as well inducers of gp96 expression which matched the elevated gp96 mRNA levels that correlated with the degree of inflammation in biopsies from IBD patients. Although a lack of expression of gp96 in macrophages is associated with CD [9], a higher overall expression in biopsies from IBD patients was detected in the present study which was consistent with the data from epithelial cells of IBD patients [9], [14]. It would be interesting to identify the cell type responsible for the elevated gp96 mRNA levels in biopsies consisting of epithelial and muscle cells, macrophages, dendritic cells and fibroblasts. However, it remains unclear whether changes in gp96 levels are cause or consequence of inflammatory processes in those patients.

After stimulation, we observed a higher change in mRNA than in protein levels of gp96. This could either mean that translation was partially inhibited or that already synthesized protein was lost because of secretion or intracellular degradation. Both processes were already considered to explain the absence of gp96 protein in IMACs from Crohn's disease patients [9]. Another possibility includes the difficulty to detect changes of abundantly expressed proteins like gp96 [12], since considerable amounts of newly synthesized protein would be needed to result in a detectable change. Accordingly in breast cancer and B cell lymphoid cells with a very low basal expression, high protein levels of gp96 upon stimulation with the cytokines IL-2 and IFN-γ were detected [43]. Since data for mRNA expression were not presented, it is impossible to judge if they observed the same discrepancy between transcription and translation as we did.

Being an essential protein during early embryogenesis – the genetic knock-out is lethal [44] – a special relevance of gp96 involved in developing inflammatory and autoimmune diseases comes from the fact that apparently changes in its intracellular levels and distribution are crucial [9], [45]. Our experimental set-up using crude extracts did not allow for either the detection of intracellular relocation or secretion of gp96. Only recently the secretion of the cytoplasmic analogue HSP90β had been reported in culture supernatants of osteosarcoma cells [46], but in general HSPs of the HSP70 group have been found to be actively secreted [47].

The regulation of gp96 gene expression depends on the promoter containing consensus sequences of various transcription factors. Which ones are finally initiating transcription is a matter of how stimuli, signalling pathways and the present subset of regulatory proteins are integrated. In this regard it was an interesting and new finding in our experiments that gp96 induced its own expression. This raises the question of the underlying physiological processes. Chronologically recognition, binding and uptake by specific receptors should precede signalling pathways and finally recruitment of the transcriptional machinery to the gp96 promoter. Three gp96-receptors are known in humans: the α_2_-macroglobulin receptor CD91 [48], the scavenger receptors SR-A (Type A) [27] and SREC-I (Type F) [28], all involved in different signalling pathways. Also an unspecific uptake has been considered as possibly relevant in the uptake of gp96 [49]. In order to get an insight into the mechanism of self-induction future studies will aim at investigating release, uptake, and subsequent intracellular distribution of gp96. This will clarify further whether the regulation of gp96 expression plays a role during CD pathophysiology.

## Materials and Methods

### Isolation of Human Peripheral Blood Monocytes

Monocytes (MOs) were isolated according to [50] using Ficoll® gradient centrifugation and labelling the interphase fraction with magnetic microbeads (Monocyte Isolation Kit II, Miltenyi Biotec, Bergisch Gladbach, Germany) and a final purification with a magnetic cell sorter according to the manufacturer's instructions (AutoMACS, Miltenyi Biotec, Germany).

### Patients

Primary human IMACs were obtained from surgical specimens from intestinal mucosa of 6 CD, 5 UC, 8 diverticulitis, 3 diverticulosis, and 36 control patients (31 with carcinoma, 3 with fistulae, 1 with volvulus, and 1 healthy patient) undergoing large or small bowel surgery. This study was approved by the Cantonal Ethics Committee of Zurich and performed according to the Declaration of Helsinki (EK: 1755). For analysis of mRNA expression in intestinal biopsies from IBD patients cDNA samples were obtained from an earlier study [51].

### Isolation of IMACs

Human IMACs were isolated from surgical specimens according to a previously established protocol [6]. Briefly, mucosa was incubated in HBSS with 1 mmol/l EDTA for 15 minutes at 37°C in order to remove intestinal epithelial cells. Subsequently, mucosa was digested in 2 ml PBS with 1 mg/ml collagenase type I ( = 336 U/ml), 0.3 mg/ml DNase (Boehringer, Mannheim, Germany), and 0.2 mg/ml hyaluronidase without fetal calf serum at 37°C for 30 minutes. The liberated lamina propria cells were submitted to Ficoll® density gradient centrifugationfor isolation of mononuclear cells. Purification of IMACs was achieved by labelling the interphase fraction with immunomagnetic microbeads armed with CD33 antibody, and magnetic cell sorting according to manufacturer's instructions (AutoMACS, Miltenyi Biotec, Germany).

### Generation of *in vitro*-differentiated Macrophages (*i.*v.MACs)

*I.*v.MACs were isolated according to a previously established protocol [52]. 1×10^6^ monocytes were cultivated in Teflon bags for one week under optimal conditions (37°C, 5% CO_2_). Generation of macrophages was achieved by addition of 2% hunan AB serum. Detachment of the cells from the Teflon bag was achieved by incubation at 4°C.

### Generation of *in vitro*-differentiated Dendritic Cells (*i.*v.DCs)

*I.*v.DCs were generated according to a previously established protocol [50]. Monocytes were cultivated in flasks for one week under optimal conditions with IL-4 (5 ng/mL) and GM-CSF (50 ng/mL) added for differentiation into *i.*v.DCs.

### Cell culture

The monocytic cell line Mono Mac-6 (MM6, obtained from DMSZ) was maintained in MM6 media according to the American Type Culture Collection (ATCC) and passaged three times a week to keep cell counts below 1×10^6^/mL.

### Stimulation of cells

Primary cells were adjusted to a density of 1×10^6^/mL, the monocytic cell line MM6 was adjusted to 5×10^5^/mL. The stimulating compounds were added for 2 hours (for total RNA isolation) and between 4 and 24 hours (for protein analysis), respectively. In order to induce ER-stress in cells tunicamycin (MM6: 0.2, 2 and 20 µg/mL, i.v.DCs: 2 µg/mL) was added for indicated times from a stock solution of 1 mg/mL in DMSO. After stimulation the cells were harvested for RNA isolation or protein extraction.

### Quantitative analysis of gene expression

Isolation of total RNA was carried out with the QIAcube® system using the silica-based RNeasy method (QIAgen, Hilden, Germany). The RNA concentration was determined with the Nanodrop spectrophotometer ND 1000 (Wilmington, Delaware, USA) and adjusted to the lowest concentrated sample within the experimental setup. The adjusted RNA was reverse-transcribed with the “High-Capacity cDNA Reverse Transcription Kit” from Applied Biosystems (Carlsbad, California, USA). Real-time quantitative PCR by TAQMAN (Applied Biosystems) was used to determine the expression of genes encoding gp96 and the house keeping genes glyceraldehyde-3-phosphate dehydrogenase or β-actin. Two-step PCR was performed and fluorescence was detected at the end of each extension step. Gene expression was calculated using the 2^−ΔΔC^_T_.

### Western blotting

Cell lysates were adjusted to a concentration of 1 mg/mL. Samples (10 to 20 µg per lane) were separated using 10% SDS-PAGE. Antibodies against gp96 (Stressgen, Ann Arbor, Michigan, USA), β-actin (Sigma, St. Louis, Missouri) and GAPDH (AbD Serotec, Oxford, UK) were used to detect the corresponding antigens. Labelled bands were detected using the Western Lightning Plus ECL detection system from Perkin Elmer (Waltham, Massachusetts, USA). For band intensity analysis OptiQuant 2.50 from Packard Instruments (Meriden, Connecticut, USA) was used.

### Statistical analysis

The statistical significance of gp96 mRNA expression levels in IBD patients was determined using ANOVA by GraphPad Prism 5.
